# The Emerging Role of miRNAs for the Radiation Treatment of Pancreatic Cancer

**DOI:** 10.3390/cancers12123703

**Published:** 2020-12-09

**Authors:** Lily Nguyen, Daniela Schilling, Sophie Dobiasch, Susanne Raulefs, Marina Santiago Franco, Dominik Buschmann, Michael W. Pfaffl, Thomas E. Schmid, Stephanie E. Combs

**Affiliations:** 1 Institute of Radiation Medicine (IRM), Department of Radiation Sciences (DRS), Helmholtz Zentrum München, 85764 Neuherberg, Germany; lily.nguyen@tum.de (L.N.); daniela.schilling@tum.de (D.S.); sophie.dobiasch@tum.de (S.D.); susanne.raulefs@helmholtz-muenchen.de (S.R.); franco.marinasantiago@gmail.com (M.S.F.); thomas.schmid@helmholtz-muenchen.de (T.E.S.); 2 Department of Radiation Oncology, School of Medicine, Technical University of Munich (TUM), Klinikum rechts der Isar, 81675 Munich, Germany; 3 Deutsches Konsortium für Translationale Krebsforschung (DKTK), Partner Site Munich, 81675 Munich, Germany; 4 Division of Animal Physiology and Immunology, TUM School of Life Sciences Weihenstephan, Technical University of Munich (TUM), 85354 Freising, Germany; dominik.buschmann@wzw.tum.de (D.B.); michael.pfaffl@wzw.tum.de (M.W.P.)

**Keywords:** pancreatic cancer, miRNA, radiotherapy, radioresistance, personalized medicine, biomarker, target

## Abstract

**Simple Summary:**

Pancreatic cancer is an aggressive disease with a high mortality rate. Radiotherapy is one treatment option within a multimodal therapy approach for patients with locally advanced, non-resectable pancreatic tumors. However, radiotherapy is only effective in about one-third of the patients. Therefore, biomarkers that can predict the response to radiotherapy are of utmost importance. Recently, microRNAs, small non-coding RNAs regulating gene expression, have come into focus as there is growing evidence that microRNAs could serve as diagnostic, predictive and prognostic biomarkers in various cancer entities, including pancreatic cancer. Moreover, their high stability in body fluids such as serum and plasma render them attractive candidates for non-invasive biomarkers. This article describes the role of microRNAs as suitable blood biomarkers and outlines an overview of radiation-induced microRNAs changes and the association with radioresistance in pancreatic cancer.

**Abstract:**

Today, pancreatic cancer is the seventh leading cause of cancer-related deaths worldwide with a five-year overall survival rate of less than 7%. Only 15–20% of patients are eligible for curative intent surgery at the time of diagnosis. Therefore, neoadjuvant treatment regimens have been introduced in order to downsize the tumor by chemotherapy and radiotherapy. To further increase the efficacy of radiotherapy, novel molecular biomarkers are urgently needed to define the subgroup of pancreatic cancer patients who would benefit most from radiotherapy. MicroRNAs (miRNAs) could have the potential to serve as novel predictive and prognostic biomarkers in patients with pancreatic cancer. In the present article, the role of miRNAs as blood biomarkers, which are associated with either radioresistance or radiation-induced changes of miRNAs in pancreatic cancer, is discussed. Furthermore, the manuscript provides own data of miRNAs identified in a pancreatic cancer mouse model as well as radiation-induced miRNA changes in the plasma of tumor-bearing mice.

## 1. Introduction

Pancreatic cancer is one of the most lethal cancers and could be the second leading cause of cancer-related deaths within the next decade [1]. The most common type of malignant pancreatic neoplasms is of ductal origin and is classified as pancreatic ductal adenocarcinoma (PDAC) [2]. Despite intense research efforts, the prognosis of patients with PDAC still remains very poor, with a five-year overall survival (OS) rate of less than 7%, without any significant improvements over the past years. The majority of patients are diagnosed in locally advanced or metastatic stages because of unspecific symptoms, a lack of early sensitive and specific markers, and difficulties in imaging early-stage tumors [2].

The only potentially curative treatment option for patients with pancreatic cancer is surgical resection. However, only 15–20% of patients are eligible for surgery at the time of diagnosis due to highly aggressive tumor growth with perineural and vascular invasion and early distant metastases [3]. Therefore, the importance of neoadjuvant treatments, including chemotherapy, radiotherapy (RT), or combined chemoradiotherapy (CRT), is evident. In particular, the aim of neoadjuvant strategies is a tumor downsizing enabling a secondary resection to improve a long-term prognosis in patients with borderline and primary non-resectable, locally advanced pancreatic cancer (LAPC) [4].

Clinical trials have shown the efficacy of RT in about 30% of pancreatic cancer patients [5]. However, international standardized therapeutic guidelines are lacking and the role of RT as a treatment option for patients with LAPC is controversially discussed in the literature [6]. Different clinical trials reported conflicting results regarding the benefit of combining RT and chemotherapy. While no benefit of subsequent RT was observed in various clinical trials when compared to chemotherapy alone [7,8], others reported an improvement in OS for LAPC patients treated with CRT [9]. Concurrent chemotherapy agents comprising capecitabine, 5-fluorouracil, or gemcitabine are used in neoadjuvant CRT treatment regimens for patients with LAPC [10]. Addition of 5-fluorouracil to RT significantly improved OS compared to RT alone [11]. Capecitabine-based CRT showed a trend toward preferable progression free survival (PFS) in comparison to gemcitabine-based CRT after induction chemotherapy for LAPC [12]. More prospective studies are needed to elucidate the benefits of associating RT with systemic therapy.

The recent focus of neoadjuvant treatment strategies for LAPC lies in investigating more effective chemotherapy schemes, such as FOLFIRINOX (leucovorin, 5-fluorouracil, irinotecan, and oxaliplatin) [13,14,15,16,17], and subsequent CRT or RT with modern techniques (e.g., stereotactic, intensity modulated, and particle RT) [18]. The high failure rate of RT in PDAC can be attributed to the high intrinsic radioresistance of the tumors [6]. Additionally, pancreatic cancer is characterized by high heterogeneity, genetic diversity, presence of a dense desmoplastic tumor stroma, cancer stem cells, and a complex tumor microenvironment. These factors contribute to a high resistance to conventional treatment options such as RT, chemotherapy, and molecularly targeted therapies [19,20].

Consideration of molecular profiles or tumor subtypes for therapy decisions is not yet implemented in clinical routine. To further increase the efficacy of CRT, novel molecular biomarkers are urgently needed to define the subgroup of pancreatic cancer patients who benefit from CRT more precisely [21].

MicroRNAs (miRNAs) are highly conserved small non-coding RNAs consisting of 20–24 nucleotides and regulate protein output and gene expression at transcriptional or post-transcriptional levels [22]. Previous studies have attributed critical roles to miRNAs in biological processes such as cell proliferation, differentiation, and survival. Furthermore, miRNAs have been implicated as crucial players during development, physiology, homeostasis, and disease, and have been shown to regulate the initiation and progression of many malignancies by controlling oncogenic and tumor-suppressive pathways [23,24]. Therefore, miRNAs could have potential as novel biomarkers for diagnosis, monitoring of recurrence, and predicting prognosis and survival of patients with pancreatic cancer [22]. A recently published review article analyzed the association between miRNAs and response to chemotherapy (gemcitabine and 5-fluorouracil) in pancreatic cancer [25]. Multiple miRNAs were identified that might contribute to chemotherapeutic resistance or sensitivity.

Our article provides an overview of the current literature concerning miRNAs as blood biomarkers and miRNAs associated with radioresistance as well as radiation-induced changes of miRNAs in pancreatic cancer. Additionally, own data identifying miRNAs in a pancreatic cancer mouse model as well as radiation-induced miRNA changes in the plasma of tumor-bearing mice are included.

## 2. Results

### 2.1. miRNAs as Biomarker in Pancreatic Cancer

It has been reported that serum and other body fluids contain stable miRNAs signatures. Circulating miRNAs in PDAC blood samples represent a valuable source of information for either defining eligible therapy options, monitoring therapeutic response or predicting prognosis [26]. In a meta-study, Chhatriya et al. published a list of in total 21 miRNAs, which are designated as a “meta-signature” of miRNAs in PDAC altered in both serum and cancer tissue [27].

Ouyang et al. showed that miR-10b levels in PDAC plasma samples are highly increased compared to healthy controls or patients with chronic pancreatitis [28]. The authors claim that miR-10b is not only suitable as a diagnostic marker but could serve as a therapeutic target by interrupting the growth-promoting deleterious EGF-TGF-β interactions and antagonizing the metastatic process. In addition, a study published in 2017 by Qu et al. described that miR-21-5p might be a stable and high-accuracy diagnostic biomarker for PDAC patients [29]. Recent studies confirmed the high discriminative impact of miR-21-5p for PDAC [30] and uncovered an association with a significant unfavorable prognostic outcome [31]. miR-221 seems to be a plasma marker for monitoring the tumor status due to its high preoperative plasma concentration and significantly reduced levels post-surgery [32]. These findings suggest that miR-221 may be released from the tumor into the bloodstream and therefore reflects tumor dynamics, which could be used for monitoring a possible tumor recurrence.

Several altered miRNA expression signatures, e.g., the downregulation of miR-141 and miR-720 activating ZEB-1 and TWIST1 transcription, have been identified to discriminate between PDAC patients with and without nodal metastasis [33]. A very recent study established a 4-miRNA signature (miR-29c, miR-125a, miR-200b, miR-155) that predicts local-regional failure and overall survival of pancreatic cancer patients who underwent tumor resection with and without chemotherapy but did not receive radiotherapy [34]. Therefore, defining the miRNA expression profile of individual tumors could improve the diagnosis, the ability to select better treatment options, e.g., aggressive treatment for patients with lymph node metastasis or adjuvant chemoradiation for patients with local-regional failure and ultimately predict the respective patient’s outcome.

The Carbohydrate antigen CA19-9 is a blood antigen, and its increased level is approved as a biomarker for pancreatic cancer [35]. CA19-9 is released from the cell surface of pancreatic cancer cells, and its serum concentration is related to tumor mass and recurrence. Nevertheless, despite its low specificity and sensitivity, it is the most common diagnostic marker in PDAC patients. Recent studies postulate that the combination of CA19-9 together with plasma miRNAs can effectively be used for screening of early tumor stages and prognostic stratification due to improved specificity and sensitivity. This strategy has already been validated by combining CA19-9 with miR-16 and miR-196a [36] or miR-33a-3p and miR-320a [30].

The study from LaConti et al. analyzed serum miRNAs in a transgenic PDAC mouse model to establish novel circulating biomarkers for PDAC progression [37]. This study uncovered that miRNA changes show remarkable similarities between pancreatic cancer in patients and a transgenic pancreatic cancer mouse model. In summary, the authors analyzed eight different miRNAs and identified two miRNAs, miR-10 and miR-155, that were increased in serum of PDAC mice compared to control mice [37].

**Figure 1:**
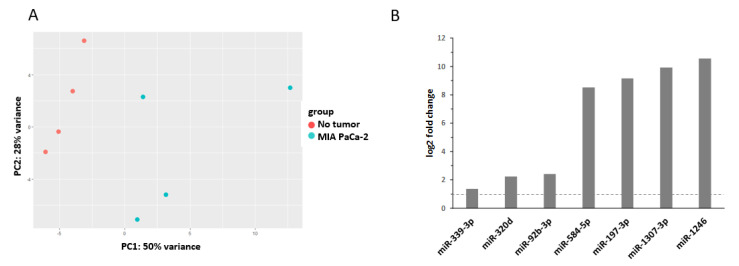
Detection of tumor-specific miRNAs in the plasma. (**A**). Principal component analysis of miRNA expression in plasma derived from non-tumor and tumor-bearing (MIA PaCa-2) mice. Analysis of all miRNAs in the dataset separates plasma samples derived from control mice (no tumor) to tumor-bearing (MIA PaCa-2) mice (PC1) and PC2 shows intra-group variability. (**B**). The upregulated miRNAs in the plasma of tumor-bearing (MIA PaCa-2) mice compared to the plasma of non-tumor-bearing mice are shown. Significant miRNAs were selected based on a log2 fold change ≥ |1| and an adjusted *p*-value of ≤0.05. Only transcripts with a base mean ≥50 were included.

In our study, we aimed to identify all tumor-specific miRNAs—without pre-selecting miRNAs—that are present in the plasma of mice harboring human MIA PaCa-2 tumors compared to non-tumor-bearing mice (supplementary methods and Table S1). Principal component analysis of miRNA expression distinguished tumor-bearing from non-tumor-bearing mice (Figure 1A). Seven miRNAs that are significantly upregulated in the plasma of tumor-bearing mice were identified (Figure 1B): miR-339-3p, miR-320d, miR-92b-3p, miR-584-5p, miR-197-3p, miR-1307-3p, and miR-1246. The predicted targets of these miRNA are summarized in Table S1.

Hereinafter, the miRNAs upregulated in plasma samples of tumor-bearing mice are discussed in the context of the recent literature and summarized in Table 1.

miR-339-3p was found to be deregulated in Vater’s papilla adenocarcinoma [38]. In addition, miR-339-3p was downregulated in colorectal cancer (CRC) and its low-level expression was associated with lymph node metastasis in patients with CRC [39].

In relationship to pancreatic disorders, miR-320d was identified as a potential marker for late chronic pancreatitis [40]. miR-320d was recognized as a promising biomarker for early diagnosis of CRC because miR-320d expression could discriminate adenoma and CRC patients from healthy controls [41]. In serum samples of hepatocellular carcinoma (HCC) patients, the expression level of exosomal serum miR-320d was remarkably reduced compared to the respective controls [42].

A study from Long et al. described reduced miR-92b-3p expression levels in human PDAC tissues and a correlation with advanced tumor/node/metastasis (TNM) stages. Moreover, it was postulated that miR-92b-3p might act as a tumor suppressor in PDAC [43]. In gastric cancer, the serum level of exosomal miR-92b-3p was found to be higher than in healthy individuals, thus serving as a potential non-invasive biomarker for early diagnosis of gastric cancer [44].

miR-584-5p was negatively associated with tumor size in gastric cancer and therefore highlighted as a tumor suppressor and potential therapeutic target [45]. In lung adenocarcinoma, miR-584-5p was one of six upregulated miRNAs which might serve as circulating biomarkers for the early tumor detection [46].

The analysis from Ni et al. showed that miR-197-3p was downregulated in HCC tissues and low levels were correlated with larger tumor size and enhanced invasion, indicating an aggressive subtype [47].

Interestingly, miR-1307-3p and miR-1246, which were also deregulated in our study (Figure 1B), are two of five miRNAs (miR-1246, miR-1307-3p, miR4634, miR-6861-5p, and miR-6875-5p) that are suitable for early detection of breast cancer [48]. In addition, the authors claim that the combination of these five serum miRNAs can be used to differentiate breast cancer from benign diseases of the pancreas, biliary tract, and prostate, or from other cancers.

Additionally, the upregulation of miR-1246 in combination with miR-196a in plasma exosomes was found to be a potential indicator for localized pancreatic cancer. Therefore, these miRNAs might serve as circulating biomarkers for the early detection of this severe disease [49]. The study from Wei et al. showed that the expression level of serum miR-1246 was upregulated in PDAC patients compared to healthy controls and strongly reduced after tumor resection [50]. In addition, miR-1246 was part of a four-miRNA panel that was significantly upregulated in the serum of pancreatic cancer patients [51]. Further studies described elevated serum exosomal miR-1246 as a potential biomarker for the early diagnosis of gastric cancer [52] as well as esophageal squamous cell carcinoma (SCC) [53] and cervical cancer [54].

The listed publications demonstrate that circulating miRNAs represent a rich source of potential biomarkers for the early diagnosis, classification, therapeutic success, and recurrence monitoring in various human cancers, especially PDAC. Therefore, miRNA-based liquid biopsy holds promising impact for further implementation in the clinical routine.

### 2.2. miRNA Response to Ionizing Radiation

Radiation therapy uses ionizing radiation (IR) to induce double-strand breaks in the genomic DNA of tumor cells. Failure to restore genomic integrity before mitosis can lead to cell death or malignant transformation. Therefore, cells trigger the DNA double-strand repair. In this complex process, first sensor proteins recognize the DNA damage. Subsequently, transducer proteins recruit effector proteins responsible for cell cycle arrest, apoptosis, transcription arrest, and DNA repair [55].

Increasing evidence demonstrates that miRNAs play a critical role in the cellular response to IR, as multiple examples of miRNA expression changes in response to IR have been reported [56,57]. It was shown that miRNAs are responsible for regulating almost every cellular pathway, including the DNA damage response (DDR), after IR [55]. Whereas some miRNAs involved in the DDR are upregulated by IR (e.g., miR-34a, miR-100, and miR-143), others (e.g., miR-15b, miR-222, and miR-181a) are downregulated [55]. Interestingly, several miRNAs participating in the DDR (e.g., let-7 family, miR-15a, miR-16, miR-21, miR-24, miR-155, miR-182, and miR-302 cluster) have been reported to be both down- and upregulated, depending on the cell type, radiation dose, and time point of measurement post-RT [55].

Conversely, different members of the DDR pathway are involved in radiation-induced miRNA regulation. Ataxia telangiectasia mutated (ATM), a DDR pathway member, plays an important role in the regulation of miRNA biogenesis [58]. ATM-dependent activation of KH-type splicing regulatory protein (KSRP) upon DNA damage increases miRNA processing and expression of a specific class of miRNAs. miR-21 and miR-16, both playing a role in DDR, belong to the miRNAs that are upregulated by ATM-KSRP. Activation of the transcription factor p53 by IR leads to enhanced expression of the miR-34 family and also regulates other miRNAs, including let-7a and let-7b [59,60]. IR also affects the activity of other transcription factors (e.g., NF-kB, Myc, and E2F), which modulate the expression of several miRNAs in the DDR [57].

For pancreatic cancer, only few in vitro studies investigated radiation-induced miRNA changes [61]. Radiation significantly reduced the levels of miR-99b, leading to enhanced mTOR expression and radioresistance [61]. Very recently Jiang et al. performed miRNA sequencing and identified miR-196b-5p and miR-194-5p to be upregulated after irradiation in exosomes derived from dying pancreatic cancer cells (SW1990, Panc-1) [62]. Furthermore, irradiation with 10 Gy induced miR-194-5p upregulation in different pancreatic cancer cell lines (SW1990, Panc-1, MIA Paca-2) [62]. miR-194-5p mimics reduced the DNA damage in irradiated pancreatic cancer cells suggesting that miR-194-5p promotes survival and radioresistance of pancreatic cancer cells [62].

A very recent systematic review and meta-analysis summarize the effects of RT on circulating miRNAs in humans, nonhuman primates, and mice [63]. Radiation treatment in the included studies was very heterogeneous, including total body irradiation and local tumor-specific irradiation with different fractionation schemes and irradiation doses. Nevertheless, the meta-analysis identified 28 miRNAs with significant radiation-induced changes (18 miRNAs upregulated and 10 miRNAs downregulated). Whereas in this meta-analysis nine publications analyzed the effect of total body irradiation on the miRNA levels in plasma/serum of healthy mice, only one study used a tumor xenograft model (breast cancer) and investigated the impact of localized radiotherapy on tumor-specific miRNA plasma levels [64]. In this xenograft mammary tumor mouse model, decreased plasma levels of miR-155, miR-10b, and miR-21 have been detected after RT [64]. Specifically, for pancreatic cancer, only one study analyzed the impact of RT on the expression level of one specific miRNA and found enhanced miR-194-5p levels in exosomes derived from the plasma of irradiated PDX (patient derived xenograft) mice [62]. No in vivo studies describing the effect of RT on the expression levels of different miRNAs in pancreatic cancer were found in the literature.

**Figure 2:**
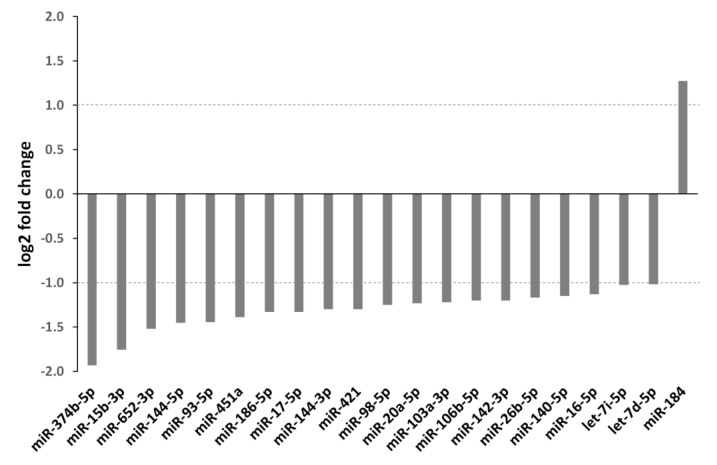
Radiation-induced changes in plasma miRNA levels. The deregulated miRNAs in the plasma of tumor-bearing (MIA PaCa-2) mice after 5 Gy irradiation of the tumor when compared with the plasma of tumor-bearing (MIA PaCa-2) mice receiving sham (0 Gy) irradiation are shown. Positive log2 fold changes indicate miRNAs upregulated in the plasma of irradiated mice compared to plasma of unirradiated mice whereas negative log2 fold changes indicate miRNAs downregulated in plasma of irradiated mice. Significant miRNAs were selected based on a log2 fold change ≥ |1| and an adjusted *p*-value of ≤0.05. Only transcripts with a base mean ≥50 were included.

Therefore, we investigated the impact of radiation on circulating miRNA levels in a pancreatic cancer xenograft mouse model (supplementary methods and Table S2). MIA PaCa-2 tumors were irradiated with a single dose of 5 Gy. Sham-irradiated (0 Gy) tumor-bearing mice served as control. Mice were sacrificed 24 h after irradiation and the plasma was collected. Small RNA sequencing revealed 21 miRNAs that were significantly modified (Figure 2). Interestingly, 20 miRNAs were down-regulated after irradiation with 5 Gy, and only one miRNA (miR-184) was upregulated. The predicted targets of these miRNAs are summarized in Table S2.

From an earlier study, it is known that miR-374b functions as an oncogene by targeting PTEN, resulting in the activation of the PI3K/Akt signaling cascade in human gastrointestinal stromal tumor cells [65]. However, there are no reports concerning the role of miR-374b in pancreatic cancer yet. It is known that the miR-15b family is involved in cell cycle regulation, proliferation, and apoptosis [66]. A recent study showed that the expression of SMURF2, a tumor suppressor gene, is inhibited by miR-15b in pancreatic cancer [67]. There is also evidence of the downregulation of miR-652 in various cancerous diseases, but its role in the radiation response is still unknown [68].

In PDAC, the overexpression of miR-144-3p reduces cell migration, proliferation, and invasion [69]. Currently, an increasing number of miR-93 and miR-106b target genes have been identified, suggesting miR-93 and miR-106b may differentially affect the behavior of tumors. A study found that the upregulation of miR-93/106b enhances PD-L1 in response to irradiation [70]. It is known that the PD-1/PD-L1 pathway serves as a mechanism for tumors to evade an antigen-specific T cell immunologic response. However, pancreatic cancer patients have been shown to be mostly resistant to PD-1 inhibition [71].

The downregulation of the tumor-suppressive miR-451a leads to enhanced cancer cell migration and invasion in hypopharyngeal SCC [72]. miR-186 overexpression has been observed in PDAC and was shown to contribute to its invasive potential [73]. Different studies revealed upregulation of miR-17 in pancreatic cancer, leading to increased proliferation and invasion [74,75]. Furthermore, inhibition of miR-17 increased the sensitivity of pancreatic cancer cell lines to chemotherapy by upregulation of Bim [76]. miR-421 has been found to be upregulated in pancreatic cancer as an oncogene and potential regulator of DPC4/Smad4 [77].

miR-98, a member of the let-7 family, regulates many cellular biological responses, including cell migration and apoptosis, after irradiation. The radiation-induced inhibition of miR-98 is closely related to both the p53 and ATM signaling pathways [78,79]. In a recent publication, it was shown that downregulation of miR-98-5p and other members of the let-7 family leads to increased PDAC proliferation and metastasis by reversely regulating the MAP4K4 pathway [80]. In the study of Morimura and colleagues, miR-20a levels in the plasma of pancreatic cancer patients were increased compared with healthy donors [81]. It was shown that the expression of miR-20a promotes radioresistance in nasopharyngeal cancer cells [82]. Higher levels of miR-20a activate the PTEN/PI3K/Akt signaling pathway and induce radioresistance of HCC [83]. A recent study identified miR-103 as one of the most important miRNAs in a functional screen for DNA damage disrupting agents. The authors identified Rad51 and Rad51D as key targets of miR-103 in tumor cells [84].

Another in vitro study demonstrated that miR-142 has a regulatory relationship with HIF-1α. miR-142 inhibits pancreatic cancer cell proliferation and invasion, partly by targeting HIF-1α at its binding site [85]. An earlier study analyzed the role of miR-26a in pancreatic tissue by quantifying its expression levels in 106 PDAC tissue samples [86]. The authors found that miR-26a expression was downregulated in PDAC compared to adjacent normal tissue. miR-140 inhibits cell viability, proliferation, and invasion in PDAC [87]. One study showed that miR-16 can enhance radiation sensitivity by regulating the TLR1/NF-κB signaling pathway and act as a potential therapeutic approach to overcome radioresistance for lung cancer treatment [88]. Jiao et al. compared the miRNA expression profile in PDAC with benign cystic tumors to identify miRNAs deregulated during PDAC development [89]. Amongst others, let-7i and let-7d were downregulated in tissue samples of PDAC [89].

The only miRNA that was significantly upregulated after irradiation in our study was miR-184. In recent years, miR-184 has been extensively explored in various cancer types. miR-184 was not only found to be upregulated in pancreatic cancer but can also facilitate the proliferation and invasion of tumor cells while suppressing apoptosis [90].

### 2.3. miRNAs and Radioresistanc

miRNAs are well known to affect the radiosensitivity by modulating DNA damage repair, cell cycle checkpoints, apoptosis, signal transduction pathways, and the tumor microenvironment [91]. Both DNA double-strand repair pathways, the non-homologous end-joining (NHEJ) and homologous recombination (HR), are targeted by miRNAs. Furthermore, miRNAs can influence signaling pathways mediating radioresistance, such as PI3-K/Akt, NF-κB (nuclear factor-kappa B), MAPK (mitogen-activated protein kinase), and TGFβ (transforming growth factor-β). Prominent examples of miRNA targets affecting radioresistance are H2AX, BRCA1, ATM, DNA-PK, RAD51, Chk1, Cdc25, and p53 [91]. Overexpression of miR-138 and miR-24 was reported to reduce the expression of H2AX and subsequently diminishes DNA repair capacity [92,93]. miR-21 targets the cell cycle checkpoint gene Cdc25 and thereby modulates radiosensitivity [94]. miR-421 and miR-101 suppress ATM expression and sensitize tumor cells to radiation [95,96].

In the following section the role of the miRNAs—that were found to be down- or upregulated by irradiation (Figure 2)—in mediating radioresistance/radiosensitivity was investigated. Whereas five of these miRNAs contribute to increased radioresistance (miR-374b, miR-93, miR-20a, miR-106b, miR-140), nine miRNAs (miR-15b, miR-451, miR-186, miR-421, miR-98, miR-142, miR-26b, miR-16, let-7 family) mediate decreased radioresistance (Table 2). Two miRNAs (mir-144, miR-17) are controversially described in the literature concerning their effect on radioresistance. For miR-652, miR-103a, and miR-184, no data regarding their impact on the radioresponse have been found in the literature.

Direct targets of the miRNAs that mediate radioresistance comprise PTEN, FOXA1, BTG3, and NPAS2. miRNAs that evoke enhanced radiosensitivity target Chk1, Wee1, ATF2, c-MET, PIM1, c-MYC, RAB14, FOXD1, MEF2D, ATM, BCL2, CD133, EphA2, Cyclin D1, and Cyclin E1. Several of these targets (Chk1, c-Myc, ATM, Bcl-2, Cyclins) are prominent key players in the DNA damage response, cell cycle control, and apoptosis [91]. Consequently, the down-regulation of these targets by the respective miRNA (miR-15b, miR-451, miR421, miR-98, miR-16) leads to increased radiosensitivity. However, for other miRNAs the interrelation between target gene expression and radioresistance/radiosensitivity is not evident. Therefore, more research in this field is essential to understand the function and regulation of these miRNAs and their targets in mediating radioresistance, and finally to implement the data in clinical routine to improve the effectiveness of RT.

### 2.4. miRNAs and Radioresistance in Pancreatic Cancer

For pancreatic cancer specifically, there are only a few publications about the interplay between miRNAs and radioresistance. The details of miRNA expression and radioresistance in pancreatic cancer are described below and summarized in Table 3.

A recent paper showed that miR-502 overexpression increased the radiosensitivity of pancreatic cancer cell lines by targeting two proteins of the classical NHEJ repair pathway, Ku70 and XLF [122]. Mechanistically, miR-502 directly inhibits the DNA double-strand repair and also attenuates the cell cycle response upon radiation.

In radioresistant pancreatic cancer cell lines that were established by repeated exposure to radiation, miR-23b expression was reduced compared to the parental cell lines [123]. Overexpression of miR-23b rendered the radioresistant cells more sensitive to radiation. Furthermore, the expression of ATG12 (Ubiquitin-like protein), a target of miR-23b, was increased in the radioresistant cells. As ATG12 is involved in vesicle formation during autophagy, enhanced ATG12 expression increased autophagy and, subsequently, radioresistance. Furthermore, an association between decreased ATG12 expression and elevated miR-23b levels was observed in human pancreatic cancer tissue, indicating that miR-23b might affect autophagy activity in pancreatic cancer cells [123]. Another study showed that miR-216a can render radioresistant pancreatic cancer cells radiosensitive by inhibiting beclin-1-mediated autophagy [124]. Additionally, in a xenograft mouse model, miR-216a expression reduced the tumor growth after irradiation.

Tomihara et al. observed that high c-Met expression is associated with lower PFS and OS in pancreatic cancer patients receiving preoperative radiochemotherapy [125]. Further investigations revealed that miR-181b is downregulated in radioresistant pancreatic cancer cell lines leading to the upregulation of the transcription factor ETS1 and the c-Met pathway [125].

mTOR expression and subsequent mTOR signaling pathways have been upregulated after RT in human pancreatic cancer biopsies [61]. In vitro, mTOR was also upregulated upon radiation and mTOR expression has been shown to contribute to radioresistance. As mTOR is a miR-99b target, down-regulation of miR-99b enhanced mTOR expression and radioresistance in vitro [61].

Increased miR-193a levels have been found in irradiated dying pancreatic cancer cells, leading to elevated miR-193a levels in surviving cells [127]. Furthermore, miR-193a promotes proliferation and repopulation of the surviving cells via TGF-β2/TGF-βRIII signaling [127]. In patient-derived xenograft mouse models, radiation in combination with miR-193 antagonist inhibited cell repopulation and metastasis, and improved the survival. These data suggest that inhibition of miR-193 might increase radiosensitivity.

Transfection with a miR-620 mimic increased the radioresistance of MIA PaCa-2 cells and also of breast and prostate cancer cell lines [128]. The tumor suppressor gene HPGD (15-hydroxyprostaglandin dehydrogenase) was identified as a target of miR-620. miR-620 overexpression leads to degradation of HPGD, enhanced prostaglandin E2 (PGE2) levels and signaling through the EP2 receptor promoting the survival and radioresistance of tumor cells.

Pancreatic cancer cell lines have reduced miR-34a expression compared to normal pancreatic ductal epithelial cells [131]. p53 has been shown to directly regulate miR-34 family members, and subsequently, the expression of miR-34 is strongly reduced in p53-mutant cancer cells [132]. miR-34 restoration in p53-mutant pancreatic cancer cells, which have initially low miR-34 levels leads to the downregulation of anti-apoptotic Bcl-2 and the Notch signaling pathway, which is involved in the maintenance of cancer stem cells [126,132,133]. Furthermore, miR-34 restoration reduces clonogenic cell growth and enhances radio- and chemosensitivity [126]. Therefore, miR-34 may restore the tumor-suppressing function of p53 in p53-deficient human pancreatic cancer cells and might constitute a new approach to treating p53-mutated pancreatic cancer.

Inhibition of Lin28 by siRNA abrogates posttranscriptional degradation of let-7a and increases radiosensitivity of AsPc-1 cells presumably through down-regulation of Kras expression [129].

Baek et al. investigated both the influence of miRNA expression on the radiosensitivity to gamma-rays and to carbon ion beam RT [130]. Overexpression of miR-374 did not affect the sensitivity to conventional gamma-ray radiation, but the sensitivity to carbon ion beam radiation was enhanced. These data suggest that miR-374 could be a potential radiosensitizer for carbon ion RT.

In summary, only a few miRNAs associated with radioresistance in pancreatic cancer have been identified so far. Therefore, comprehensive studies analyzing miRNA expression profiles are necessary to identify miRNAs, which can predict the individual radiosensitivity or constitute targets for radiosensitization.

## 3. Discussion

Nowadays, early diagnosis is essential for the successful treatment of pancreatic cancer, and surgical resection is the only potentially curative therapy [3]. Since pancreatic cancer is a locally invasive as well as a systemic disease, the disease recurs in most patients, which leads to a five-year survival rate of only 30% after complete resection [134]. miRNAs could help to detect pancreatic cancer in an earlier stage, leading to increased curative treatments. In addition, more research into neoadjuvant treatment options, including RT or combined CRT, is urgently needed. The goal of RT is mainly to downsize the tumor to enable a secondary resection. Therefore, neoadjuvant RT may lead to improved long-term prognosis in patients with borderline and primary non-resectable, locally advanced pancreatic cancer [4]. While RT was historically a central treatment component, its role has been called into question based on the publication of clinical trials with conflicting results and due to the effectiveness of combined chemotherapeutic regimes (e.g., FOLFIRINOX) [135].

Novel molecular biomarkers are required to increase the efficacy of RT and to define which patients benefit most from it [21]. Especially, miRNAs could have the potential as novel biomarkers for diagnosis, monitoring of recurrence, and predicting prognosis and survival of patients with pancreatic cancer [22,136]. Our present study provides an identification of miRNAs in an in vivo pancreatic cancer mouse model. A panel of seven miRNAs was significantly upregulated in plasma of tumor-bearing mice (Figure 1). In the next step, this biomarker panel should be validated in plasma of pancreatic cancer patients. Especially, circulating miRNAs represent a valuable source and are easily accessible [26].

Several miRNAs have been shown to play a role in radioresistance in different tumor entities [56,91]. Furthermore, significant changes in the expression levels of miRNAs can be observed after exposure to IR. It is known that miRNAs are responsible for regulating almost every cellular pathway, including the DNA damage response after IR [57]. Understanding the regulation and function of miRNAs is essential to improve the effectiveness of RT. To determine radiation-induced miRNA changes in the plasma of tumor-bearing mice, local irradiation of subcutaneous MIA PaCa-2 tumors was performed. Small RNA sequencing revealed 21 miRNAs that were significantly regulated (Figure 2). Interestingly, 20 out of these 21 miRNAs were downregulated. Although a mechanistic basis is not yet available, the reduced levels can be assumed to promote translation of specific miRNA target proteins [137]. A current literature search reveals an association between the expression of these miRNAs, their targets (e.g., Chk1, c-Myc, ATM and Cyclins) and either radioresistance or radiosensitivity (Table 2) [98,107,113,120]. Most of these miRNAs (miR-15b, miR-451, miR-186, miR-421, miR-98, miR-142, miR-26b, miR-16, let-7 family) mediate radiosensitivity [98,105,106,107,108,109,112,113,114,116,117,118,120,121] suggesting that the radiation-induced downregulation of these miRNAs might enhance radioresistance.

Specifically, for pancreatic cancer, only very few publications about the interplay between miRNAs and radioresistance exist, which were reviewed in this manuscript (Table 3).

Although radioresistance is rather complex, emerging evidence has demonstrated that epigenetic alterations, including miRNA changes, play important roles in resistance to RT. Therefore, more comprehensive preclinical and clinical studies analyzing miRNA expression profiles in context with radioresistance are urgently needed to identify miRNAs that can predict the individual radiosensitivity. Moreover, further studies are required to confirm the molecular mechanisms of the deregulated circulating miRNAs as diagnostic biomarkers for pancreatic cancer. In addition, miRNAs can help to develop innovative cancer therapies, which is of great significance for improving the life expectancy of pancreatic cancer patients [138].

The possibility to detect miRNAs in blood samples makes them a promising tool for rapid PDAC diagnosis, as well as patient stratification, to allow personalized therapy [139]. However, miRNAs are not yet routinely used for cancer management, and we should consider the challenges behind this strategy. One of them is the need to standardize protocols [140]. From sample collection and preparation to miRNA detection, inter-laboratory reproducibility must be ensured. In this regard, sampling tubes, patient-related factors, sample storage, and sample processing can affect RNA yield and purity [141]. The high heterogeneity of PDAC should also be regarded as an additional challenge, as it makes it harder to select reliable biomarkers [139]. Concerning miRNAs applications in RT, it should also be considered that there is still insufficient knowledge about their functions in different RT concepts such as dose fractionation. Information is also lacking regarding the use of alternative radiation qualities such as protons or carbon ions and the tumor microenvironment such as hypoxia. Additionally, the translation of preclinical data from in vitro or xenograft models into clinical routine using patient materials is still poorly realized [137]. Preclinical miRNA candidates will have to be investigated in large prospective trials of different PDAC patient populations to gain confidence in the selected biomarkers [142]. Future trials should be designed to evaluate miRNA biomarkers in a temporal manner, by collecting samples before and after RT, leading us towards personalized RT for PDAC [142].

## 4. Conclusions

In the last decade, miRNAs have become promising tools as prognostic and diagnostic biomarkers as well as therapeutic targets for innovative and personalized cancer treatment. Several miRNAs have been found differentially expressed and to be predictive for the treatment outcome in multiple cancer entities. With a five-year survival rate of less than 7%, pancreatic cancer presents an urgent need to identify the subset of patients which could benefit from treatment intensification and to establish novel individualized CRT treatment options. In this article, we focused on the role of miRNAs in pancreatic cancer, radioresistance, and radiation-induced changes, which could lead the way to personalized treatment in the future.

## Figures and Tables

**Table 1:** Overview of miRNAs found to be deregulated in our analysis of plasma samples of tumor-bearing mice and associated publications.

miRNA	(Tumor) Entity	Expression	Source	Reference
miR-339-3p	Vater’s papilla adenocarcinoma	downregulated	tissue	[38]
	colorectal cancer	downregulated	tissue	[39]
miR-320d	chronic pancreatitis	upregulated	tissue	[40]
	colorectal cancer	downregulated	plasma and tissue	[41]
	hepatocellular carcinoma (HCC)	downregulated	serum exosomes	[42]
miR-92b-3p	pancreatic ductal adenocarcinoma (PDAC)	downregulated	tissue	[43]
	gastric cancer	upregulated	serum exosomes	[44]
miR-584-5p	gastric cancer	downregulated	tissue	[45]
	lung cancer	upregulated	plasma	[46]
miR-197-3p	HCC	downregulated	tissue	[47]
miR-1307-3p	breast cancer	upregulated	serum	[48]
miR-1246	breast cancer	upregulated	serum	[48]
	PDAC	upregulated	plasma exosomes	[49]
	PDAC	upregulated	serum	[50]
	PDAC	upregulated	serum exosomes	[51]
	gastric cancer	upregulated	serum exosomes	[52]
	esophageal squamous cell carcinoma (SCC)	upregulated	serum	[53]
	cervical cancer	upregulated	serum	[54]

**Table 2:** miRNAs from Figure 2 and their association with radioresistance. miRNAs, their effect on radioresistance (increased or decreased), the entity, the identified target genes, and proteins/pathways.

miRNA from Figure 2	miRNA in Reference	Radio-Resistance	Entity (In Vitro/In Vivo) *	Targets ^#^	Proteins/ Pathways ^§^	Ref.
miR-374b-5p	miR-374b-5p	increased	Canine oral melanoma (in vitro)	PTEN ^1^		[97]
	
miR-15b-3p	miR-15b	decreased	Breast cancer (in vitro)	Chk1, Wee1 ^1^		[98]
	
miR-144-5p/ miR-144-3p	miR-144-5p	decreased	NSCLC (in vitro/in vivo)	ATF2 ^1^		[99]

	miR-144-3p	decreased	Glioblastoma (in vitro)	c-MET ^1^	Phosphorylation of STAT3, ERK1/2, AKT, mTOR (all down) ^4^	[100]

	miR-144	increased	Breast cancer (in vitro)		PTEN (down) ^4^ AKT, Snail, N-cadherin, Vimentin (all up) ^4^	[101]
	miR-144	decreased	Prostate cancer (in vitro)	PIM1 ^1^		[102]
miR-93-5p	miR-93-5p	increased	Colorectal cancer(in vitro)	FOXA1 ^1^	TGFB3 (up) ^4^	[103]
	miR-93	increased	Esophageal squamous carcinoma (in vitro)	BTG3 ^1^		[104]


miR-451a	miR-451a	decreased	Mouse colorectal cancer (in vitro)	CAB39, EMSY, MEX3C, EREG ^2^		[105]
	miR-451	decreased	NSCLC (in vitro)		PTEN (up) ^4^	[106]
	miR-451	decreased	Lung adenocarcinoma (in vitro)	c-MYC ^1^	Survivin, rad-51 (both down) ^4^	[107]

	miR-451	decreased	Nasopharyngeal carcinoma (in vitro)	RAB14 ^1^		[108]

miR-186-5p	miR-186	decreased	Nasopharyngeal carcinoma (in vitro)	FOXD1 ^1^		[109]
	
miR-17-5p	miR-17-5p	decreased	Esophageal adenocarcinoma (in vitro)	PRKACB, C6orf120 ^3^	PRKACB, C6orf120 (both down) ^5^	[110]
	
	
	miR-17-5p	increased	Oral squamous cell carcinoma (in vitro/in vivo)		p21, p-p53, TNF RI, FADD (all down) ^4^ cIAP1, HIF-1α, TRAIL R1 (all up) ^4^	[111]
miR-421	miR-421	decreased	Glioma (in vitro)	MEF2D ^1^		[112]
	miR-421	decreased	Cervix carcinoma, NSCLC and SCCHN (in vitro)	ATM ^1^		[113]

miR-98-5p	miR-98	decreased	Esophageal squamous cell carcinoma (in vitro)	BCL2 ^1^		[114]
	
	
miR-20a-5p	miR-20a-5p	increased	Nasopharyngeal cancer (in vitro)	NPAS2 ^1^	Notch pathway (down) ^6^	[82]
	
miR-106b-5p	miR-106b	increased	Prostate cancer (in vitro)		p21 (down) ^4^	[115]

miR-142-3p	miR-142-3p	decreased	Breast cancer (in vitro)		β-catenin (down) ^4^	[116]
	miR-142-3p	decreased	Umbilical cord blood mononuclear cells (in vitro)	CD133 ^1^		[117]


miR-26b-5p	miR-26b-5p	decreased	Hepatocellular carcinoma (in vitro)	EphA2 ^1^		[118]
	
miR-140-5p	miR-140	increased	Lung fibroblasts (in vitro/in vivo)			[119]

miR-16-5p	miR-16-5p	decreased	Prostate cancer (in vitro)	Cyclin D1, Cyclin E1 ^1^	pRb, E2F1 (both down) ^4^	[120]

let 7i-5p let 7d-5p	let-7 family (let-7e)	decreased	Colorectal cancer (in vitro)		IGF-1R (down) ^4^	[121]

* If not otherwise indicated, human. ^#^ Targets: all down-regulated by respective miRNA, ^1^ identified by luciferase assay, ^2^ identified by miR-TRAP assay, ^3^ predicted. ^§^ Proteins/pathways regulated by respective miRNA, ^4^ demonstrated by Western Blot, ^5^ demonstrated by RT-PCR, ^6^ demonstrated by signaling pathway assay.

**Table 3:** miRNAs associated with radioresistance in pancreatic cancer. miRNAs, their effect on radioresistance (reduced or increased), the used cell lines, the identified target genes, and the intervention are listed.

miRNA	Radioresistance	Cell Lines	Targets	Intervention	Ref.
miR-502	reduced	Mia PaCa-2, PaTuT, PaTu02	Ku70, XLF	miR-502 overexpression	[122]
miR-23b	reduced	Panc-1, BxPc3	ATG12	miR-23b mimic/inhibitor	[123]
miR-216a	reduced	Panc-1, BxPc3	beclin-1	miR-216a mimic	[124]
miR-99b	reduced	Panc-1, BxPc3, Capan-2	mTOR	miR-99b precursor/inhibitor	[61]
miR-181b	reduced	Panc-1, MIA PaCa-2	ETS (c-Met)	miR-181b precursor	[125]
miR-34	reduced	BxPc3, MIA PaCa-2	Bcl-2, Notch1-2	miR-34 mimic	[126]
miR-193a	increased	Panc-1, SW1990, AsPc-1	TGF-β2/TGF-βRIII, E2F6	miR-193a antagonist	[127]
miR-620	increased	MIA PaCa-2	HPGD	miR-620 mimic	[128]
Let-7a	reduced	AsPc-1	K-Ras	Lin28 siRNA (repressor of let-7a)	[129]
miR-374	unchanged/ reduced	Panc-1, MIA PaCa-2	-	miR-374 overexpression	[130]

## Supplementary Materials

The following are available online at https://www.mdpi.com/2072-6694/12/12/3703/s1, Supplementary Methods, Table S1: miRNAs significantly upregulated in the plasma of tumor-bearing mice, Table S2: miRNAs significantly down- or upregulated in plasma of tumor-bearing mice after irradiation with 5 Gy.
