# Supplementary material for: The Emerging Role of miRNAs for the Radiation Treatment of Pancreatic Cancer
Source: Cancers (Basel). 2020 Dec 9;12(12):3703. doi: 10.3390/cancers12123703 (PMC7763922; doi:10.3390/cancers12123703)
Supplement: Supplementary file 1 [file cancers-12-03703-s001.pdf]

Supplementary material: The Emerging Role of miRNAs for the Radiation Treatment of Pancreatic Cancer

Lily Nguyen, Daniela Schilling, Sophie Dobiasch, Susanne Raulefs, Marina Santiago Franco, Dominik Buschmann, Michael W. Pfaffl, Thomas E. Schmid and Stephanie E. Combs

Supplementary Methods

Animal and Tumor Model

All animal experiments were approved by the government of Upper Bavaria, Germany, and conducted according to the German animal protection guidelines.

The established human pancreatic cancer cell line MIA PaCa-2 obtained from the German Collection of Microorganisms and Cell Cultures (DSMZ GmbH, Braunschweig, Germany) was used for a subcutaneous xenograft tumor mouse model. 4×10^6 MIA PaCa-2 cells suspended in phosphate-buffered saline (PBS) were injected subcutaneously into the right flank of 7–8 week old, female athymic CD1-Foxn1 nude mice (Charles River Laboratories, Sulzfeld, Germany).

Radiation treatment of tumor-bearing mice was initiated when tumors reached a size of 60–100 mm^3, at which point mice were randomized into the untreated or radiotherapy treatment group. Mice were locally irradiated with 5 Gy at 220 kV and 13 mA with a dose rate of 2.6 Gy/min using the Small Animal Radiation Research Platform (SARRP, XStrahl Ltd., Camberley, UK), a high-precision small animal irradiator equipped with a cone-beam CT scanner. Mice which did not receive a tumor injection served as "no tumor" control group.

Mice were immobilized with inhaled isoflurane anesthesia at a concentration of 1.5 % with 4 % volume of oxygen during tumor cell injection, imaging, treatment planning and irradiation. 24 hours after irradiation, mice were sacrificed. Blood from mice was taken by cardiac puncture and collected in EDTA tubes. The tubes were centrifuged and plasma was collected and stored at −80° C.

miRNA Sequencing

RNA from 100 µL plasma was isolated using the miRNeasy Serum/Plasma Kit (Qiagen, Hilden, Germany) and used for small RNA library preparation with the NEBNext Multiplex Small RNA Library Prep Kit from Illumina (#E7560S, New England Biolabs) as described in Buschmann et al. [1]. The samples were sequenced on Illumina HiSeq 2500 system (Illumina, San Diego, CA, USA) with the following conditions: rapid run, 50-bp single-end reads sequencing. Clustering and sequencing were performed using the HiSeq SR Rapid Cluster Kit v2 (#GD-402-4002, Illumina) and HiSeq Rapid SBS Kit v2 (#FC-402-4022, Illumina). Plasma samples of at least four mice from each treatment group were sequenced.

Sequencing data were processed as described previously [2]. FastQC (v0.10.1) was used for quality assessment of sequence length distribution. After removing sequences shorter than 15 nucleotides from the data set, remaining reads were trimmed for 3′ adapters and aligned to human miRNA sequences in the most recent version of miRbase.

miRNA Expression Analyzes

Differential gene expression analysis was performed via the Bioconductor Package DESeq2 (v1.8.1) [3]. Significant miRNAs were selected based on a log2 fold change $\geq |1|$ and an adjusted p-value of ≤0.05. Only transcripts with a base mean ≥50 were included in the analysis. Visualization of gene expression and principal component analysis were carried out in R using the packages gplots, ggfortify and RColorBrewer. miRDB was used to predict mRNAs targeted by the respective miRNAs [4,5]. Predicted target genes with a miRDB target score ≥ 99 were included in the tables.

Table S1. miRNAs significantly upregulated in the plasma of tumor-bearing mice. Significant miRNAs were selected based on a log2 fold change ≥|1| and an adjusted p-value (padj) of ≤0.05. Only transcripts with a base mean ≥50 were included. Predicted target genes with a miRDB target score ≥ 99 are shown.

miRNA	Base Mean	Log2 Fold Change	Padj	Predicted Targets (miRDB Target Score ≥ 99)
miR-339-3p	50	1.38	0.033	-
miR-320d	127	2.24	0.001	YOD1, CDKL5, ONECUT2, SPOPL, SH2B3, PLPPR1, KITLG
miR-92b-3p	211	2.41	0.001	B3GALT2, MAN2A1, FBXW7, G3BP2, FNIP1, CD69, SLC12A5, MYO1B, FBN1, EFR3A, SLC17A6, GLRA1, BTG2, MAP2K4, PIKFYVE, HIPK3, DOCK9, ITGAV, KLF4, RAB23, C21orf91
miR-584-5p	60	8.52	<0.001	-
miR-197-3p	57	9.17	<0.001	-
miR-1307-3p	96	9.93	<0.001	-
miR-1246	6872	10.57	<0.001	FAM53C

Table S2. miRNAs significantly down- or upregulated in plasma of tumor-bearing mice after irradiation with 5 Gy. Significant miRNAs were selected based on a log2 fold change ≥|1| and an adjusted p-value (padj) of ≤0.05. Only transcripts with a base mean ≥50 were included. Predicted target genes with a miRDB target score ≥ 99 are shown.

miRNA	Base Mean	Log2 Fold Change	Padj	Predicted Targets (miRDB Target Score ≥ 99)
miR-374b-5p	864	-1.93	0.025	PRDM11, PARP8, UBE3A, HIBADH, CADM2, N4BP2, ACVR2B, GABRG1, ZNF423, EN1, ADD3, NHLRC2, NETO1
miR-15b-3p	362	-1.76	0.030	-
miR-652-3p	508	-1.52	0.045	-
miR-144-5p	579	-1.45	0.030	-
miR-93-5p	3386	-1.45	0.029	ENPP5, FYCO1, DYNC1LI2, ZNFX1, NPAT, MED12L, TBC1D20, ZNF800, NPAS2, SAR1B, BRMS1L, ANKRD52, VLDLR, TXNIP, KCNB1, CLOCK, RUFY2, ARHGAP12, STK17B, PDCD1LG2, ZFYVE26, RAB22A, SLC40A1, GPR137C, REEP3, RRAGD, TBC1D9, SACS, PKD2, ZNF827, MAP3K2, NAPEPLD
miR-451a	178503	-1.39	0.038	-
miR-186-5p	3608	-1.33	0.029	RUFY3, ZC3H11A, ZNF644, REEP3, GABRA4, BMPR1A, TBL1XR1, OTUD4, RIMS2, STK17B, OSBPL8, TUT4, HOOK3, GCC2, RB1CC1, MAP2, SORT1, TRAPPC8, HNMT, NEGR1, C5orf24, TMF1, BRWD3, MAP3K2, TEAD1

miR-17-5p	798	-1.33	0.029	ENPP5, FYCO1, DYNC1LI2, ZNFX1, NPAT, MED12L, TBC1D20, ZNF800, NPAS2, SAR1B, BRMS1L, ITGB8, ANKRD52, VLDLR, TXNIP, KCNB1, CLOCK, RUFY2, ARHGAP12, KCNK10, STK17B, PDCD1LG2, ZFYVE26, RAB22A, SLC40A1, GPR137C, REEP3, RRAGD, TBC1D9, PTPN4, AAK1, SACS, PKD2, ZNF827, MAP3K2, NAPEPLD
miR-144-3p	1796	-1.30	0.031	UBE2D1, TNPO1, GABRA1, RIN2, ARID1A, UBR3, NFE2L2, ZFP36L2, ARID2, BBX, ATP1B1, TEK, MAPK6, FNDC3A, TET2, SLC20A2, FZD6, MYEF2, RARB, ADAMTS5, SLC12A2, FBN2, FUCA2, XYLT1, TOGARAM1, NACC2
miR-421	447	-1.30	0.029	TOMM70, MBD2
miR-98-5p	470	-1.25	0.022	ARID3B, LIN28B, IGDCC3, NR6A1, HMGA2, C14orf28, IGF2BP1, STARD13, TRIM71, PRTG, FIGNL2, FRMD4B, NPHP3, PTAFR, SMARCAD1, FIGN, GATM
miR-20a-5p	1114	-1.23	0.031	ANKRD52, ZFYVE26, ENPP5, GPR137C, FYCO1, DYNC1LI2, ZNFX1, PKD2, NAPEPLD, NPAT, ARID4B, MED12L, TBC1D20, ZNF800, NPAS2, FAM45A, SAR1B, BRMS1L, GPR6, ITGB8, VLDLR, TXNIP, KCNB1, CLOCK, RUFY2, ARHGAP12, KCNK10, STK17B, PDCD1LG2, RAB22A, USP46, C2CD2, SLC40A1, EZH1, ITPRIPL2, REEP3, RRAGD, TBC1D9, PLEKHA3, CFL2, PTPN4, AAK1, SACS, ZNF827, MAP3K2
miR-103a-3p	1712	-1.22	0.040	DICER1, SCN8A, AGFG1, KIF21A, NPAS3, MED26, ANO3
miR-106b-5p	701	-1.20	0.041	ENPP5, GPR137C, FYCO1, ANKRD52, DYNC1LI2, ZNFX1, PKD2, NAPEPLD, ZFYVE26, NPAT, RAB22A, ARID4B, USP46, MED12L, TBC1D20, C2CD2, ZNF800, SLC40A1, EZH1, NPAS2, FAM45A, SAR1B, ITPRIPL2, BRMS1L, GPR6, REEP3, ITGB8, RRAGD, VLDLR, TXNIP, TBC1D9, KCNB1, PLEKHA3, CLOCK, CFL2, PTPN4, AAK1, RUFY2, ARHGAP12, SACS, ZNF827, MAP3K2, KCNK10, STK17B, PDCD1LG2

miR-142-3p	929	-1.20	0.032	ZEB2, TASOR2, RICTOR, RHOBTB3
miR-26b-5p	7210	-1.17	0.045	STRADB, TET2, CASZ1, FAM98A, CDK8, SLC7A11, OTUD4, NAB1, CHORDC1, SLC2A13, CREBRF, CIPC, TET1, B3GNT5, STK39, LEF1, USP9X, TP53INP1, PTEN, KLHL42, E2F7, RPS6KA6, ETNK1, POLR3G, PITPNC1, RHOQ, OSBPL11, TNRC6B, NAP1L5, ULK2
miR-140-5p	1162	-1.15	0.029	SEPT2, ZNF800, EPB41L2
miR-16-5p	6975	-1.13	0.031	PAPPA, FASN, UNC80, FGF2, TNRC6B, PTPN4, PHF19, DESI1, UBE2Q1, LSM11, NECTIN1, GAREM1, ANKUB1, FBXO21, CCNE1, ATG14, LUZP1, SLC13A3, ARIH1, MGAT4A, EPHB2, BTRC, SPRYD3, ARL2, CASK, NUP50, DCLK1, CYB561A3, ZBTB46, FGF7
let-7i-5p	77013	-1.03	0.029	FIGNL2, NR6A1, TRIM71, IGF2BP1, IGDCC3, C14orf28, ARID3B, HMGA2, PRTG, LIN28B, STARD13, FRMD4B, PTAFR, FIGN, NPHP3, SMARCAD1
let-7d-5p	5595	-1.02	0.030	LIN28B, HMGA2, TRIM71, PRTG, STARD13, IGF2BP1, IGDCC3, NPHP3, FIGNL2, SMARCAD1, FRMD4B, FIGN, C14orf28, NR6A1, ARID3B
miR-184	109	1.27	0.042	-

References

1. Buschmann, D.; Kirchner, B.; Hermann, S.; Marte, M.; Wurmser, C.; Brandes, F.; Kotschote, S.; Bonin, M.; Steinlein, O.K.; Pfaffl, M.W., et al. Evaluation of serum extracellular vesicle isolation methods for profiling miRNAs by next-generation sequencing. *J. Extracell Vesicles.* **2018**, *7*, 1481321, doi:10.1080/20013078.2018.1481321.
2. Spornraft, M.; Kirchner, B.; Haase, B.; Benes, V.; Pfaffl, M.W.; Riedmaier, I. Optimization of extraction of circulating RNAs from plasma--enabling small RNA sequencing. *PloS one* **2014**, *9*, e107259, doi:10.1371/journal.pone.0107259.
3. Love, M.I.; Huber, W.; Anders, S. Moderated estimation of fold change and dispersion for RNA-seq data with DESeq2. *Genome Bio.* **2014**, *15*, 550, doi:10.1186/s13059-014-0550-8.
4. Chen, Y.; Wang, X. miRDB: an online database for prediction of functional microRNA targets. *Nucleic Acids Res.* **2020**, *48*, D127–D131, doi:10.1093/nar/gkz757.
5. Liu, W.; Wang, X. Prediction of functional microRNA targets by integrative modeling of microRNA binding and target expression data. *Genome Bio.* **2019**, *20*, 18, doi:10.1186/s13059-019-1629-z.

© 2020 by the authors. Submitted for possible open access publication under the terms and conditions of the Creative Commons Attribution (CC BY) license (http://creativecommons.org/licenses/by/4.0/).
